# The relationship between facial skeletal class and expert-rated interpersonal skill: an epidemiological survey on young Italian adults

**DOI:** 10.1186/1471-244X-6-41

**Published:** 2006-10-10

**Authors:** Andrea Senna, Domenico Abbenante, Lucio Tremolizzo, Guglielmo Campus, Laura Strohmenger

**Affiliations:** 1 Dept. of Medicine, Surgery and Dentistry, University of Milano; Dental Building, Via Beldiletto, 1 – 20124 Milano, Italy; 2 WHO Collaborating Centre for Epidemiology and Community Dentistry of Milano, Italy; 3 Guidonia Aeromedical Selection Centre, (RM), Italy; 4 D.N.T.B., University of Milano-Bicocca, Monza (MI), Italy; 5 Dental Institute, University of Sassari, Italy

## Abstract

**Background:**

The facial region plays a major role in determining physical attractiveness, so we assessed the hypothesis that the capability of successfully managing interpersonal relationships in young adults might be related to the facial skeletal class.

**Methods:**

1,014 young subjects applying to the Military Academy of Pozzuoli, Italy, were enrolled and the cephalometric evaluation was performed by calculating the angular relationships between skeletal points localized by the lateral cephalogram of the face, sorting the subjects in three groups corresponding to each major facial skeletal class. Concurrently, the subjects were evaluated by a team of psychiatrists administering the MMPI-2 test followed by a brief colloquium with each candidate, in order to identify those subjects characterized by low skills for managing interpersonal relationships.

**Results:**

According to the psychiatric evaluation about 20% of the subjects were considered potentially unable to manage successfully interpersonal relationships (NS). Males displayed an about two-fold increased risk of being NS. No differences were shown in the distribution of the NS male subjects among the three different facial skeletal classes. On the other hand, NS females displayed a different distribution among the three facial skeletal classes, with a trend of about two-fold and four-fold, respectively, for those subjects belonging to classes II and III, respect to those belonging to class I.

**Conclusion:**

Females may be more sensitive to physical factors determining beauty, such as the facial morphology certainly is. This finding appears to be interesting especially when thinking about possible orthodontic interventions, although further study is certainly needed to confirm these results.

## Background

Physical attractiveness has been shown to play a major role in determining both one person's self-esteem and his/her capability of successfully managing social relationships (Patzer 1996; Dion et al. 1972; Miller 1970; Berscheid and Walster 1972). Moreover, attractive individuals are more frequently regarded as desirable friends and they are considered to be socially skilled. Interestingly, in a study conducted by Efran (1974), attractive individuals were more easily judged as less guilty in a simulated jury task, and less severe punishment were recommended.

The influence of dento-facial appearance on social attractiveness has been extensively studied. For example, Shaw and colleagues, among others, clearly demonstrated that dental arrangement plays a major role in determining the perceived beauty and success of one person (Shaw et al. 1985; Shaw et al. 1980; Shaw 1981; Kerosuo et al. 1995; Sergl and Stodt 1970).

However, it is conceivable that the facial skeletal structure has a greater esthetic and physiologic impact on one subject than dental morphology alone (Jefferson 1996). As a matter of fact, Michiels and Sather (1994) previously reported that a sample of white women was judged as less attractive if displaying increased vertical features, or convex, or facial class II tendency profiles.

Therefore, our aim in this study was to investigate if a particular facial skeletal class was somehow related to the capacity of successfully managing interpersonal relationships in a population of young Italian adults, since both parameters have been shown to be related to the perceived beauty. Possibly this study might help to better understand the determinants of attractiveness, resulting useful when planning specific orthodontic and/or facial surgery interventions.

## Methods

**Figure 1:**
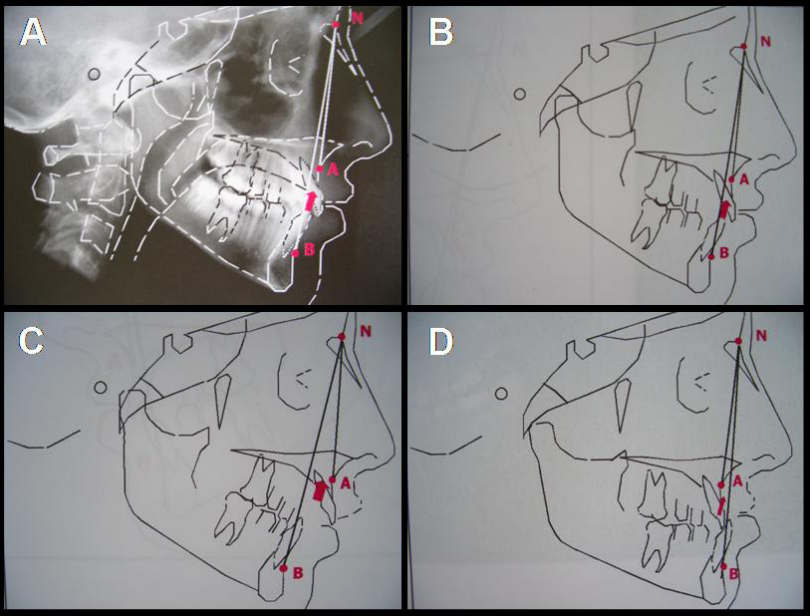
(*A*) Representative lateral cephalogram with the calculation of the ANB angle. The subject in the picture displays a I facial skeletal class, as also shown in the corresponding diagram. (*B*, *C*, *D*) Schematic representation and calculation of the ANB angle of a I, II and III facial skeletal class, respectively. Modified with permission from: "Analisi Cefalometrica e Diagnosi Ortodontica" di P.Cozza, F.Ballanti – Ed. Società Editrice Universo, Roma, 2004.

1,014 consecutive subjects (M/F: 776/248; mean age ± SD: 19.8 ± 2.5 y.o.) applying to the military academy of Pozzuoli, Italy, were enrolled following explanation of the aims of the study and obtaining informed consent. Each candidate underwent to a complete dental evaluation by two dentists, trained in the WHO Collaborating Centre for Epidemiology and Community Dentistry of Milan, Italy. No one of the enrolled subjects underwent to a previous maxillo-facial surgical intervention. The skeletal class determination was performed by calculating the angular relationship between nasion (N), sellion (S), and Down's points A and B, obtained from the lateral cephalogram (Figure 1A). Subsequently, the skeletal classes were defined on the basis of the ANB angle as follows: ANB = 2 ± 2, I class; ANB > 4, II class; ANB < 0, III class (Morabito et al. 1982; for a representation of the three facial skeletal classes see Figure 1B to 1D).

The evaluation of the capacity of successfully managing interpersonal relationships, considered to be a necessary requisite for the admission to a stressful environment, such as the Military Academy, was performed by a team of board-qualified psychiatrists. It is well known that for various reasons, individuals undergoing a psychological assessment may over-report or underreport problems. Moreover, as the stakes involved in a psychological evaluation increase, so does the likelihood that the individual will distort his or her responses (Sellbom et al. 2005). Hence, the military academy of Pozzuoli, Italy, requires every candidate to be evaluated by self-administering a standardized personality test, the Minnesota Multiphasic Personality Inventory-2 (MMPI-2; Sellbom et al. 2005), followed by a brief colloquium with the team of board-qualified psychiatrists, in order to confirm the orientation given by the test. According to the psychiatric evaluation each subject was judged either as potentially either successful (S), or not-successful (NS) in managing interpersonal relationships.

Statistical analysis was performed by using EpiInfo™ 3.3. Differences among classes were calculated by the χ^2 ^test. Linear trends in proportion were tested using χ^2 ^test for trend (Mantel, 1963).

## Results

The distribution of the recruited subjects according to the three facial skeletal classes is reported in Table 1.

**Table 1:** Distribution of male and female individuals among the three different facial skeletal classes

	**I Class ***n (%)*	**II Class ***n (%)*	**III Class ***n (%)*	**Total ***n (%)*
**M**	526 (68.67)	102 (13.32)	138 (18.01)	766 (100)
**F**	186 (75.00)	40 (16.13)	22 (8.87)	248 (100)
**Total**	712 (70.22)	142 (14.00)	160 (15.78)	1,014 (100)

Percentages refer to the distribution of either male (M), or female (F), or all (Total) subjects among the three different facial skeletal classes.

**Figure 2:**
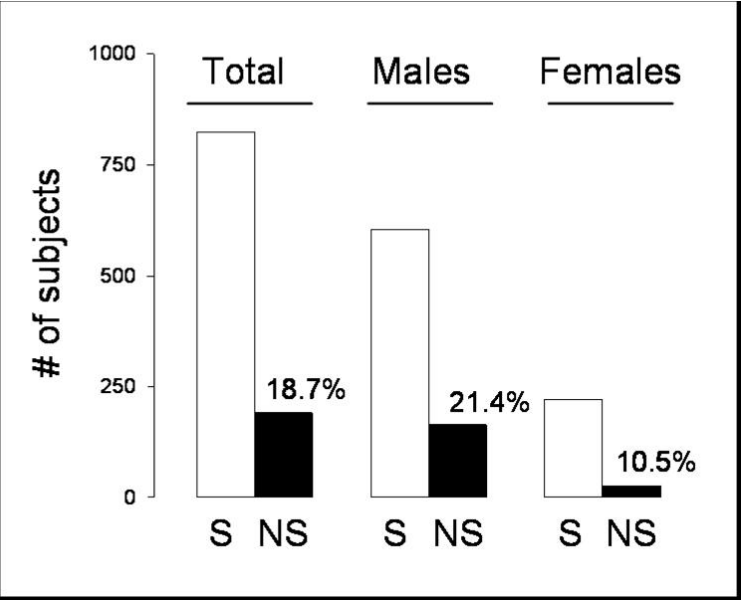
Number of subjects which were judged either as potentially succesfssul (S, white bar), or potentially not-successful (NS, black bar) in managing interpersonal relationships according to the psychiatric evaluation. The percentage of NS individuals on the total number of subjects included in each group is displayed over the corresponding black bar.

According to the evaluation of the psychiatrists about 20% of the subjects were discharged since they were potentially not-successful (NS) for managing interpersonal relationships. Male subjects displayed an about two-fold increased risk of being NS with respect to the other sex (χ^2 ^14.69, p < 0.0002; odds ratio = 2.33, 95% confidence interval: 1.47 – 3.71; see Figure 2).

The analysis of the contingency table failed to show a difference in the distribution of NS subjects among the three facial skeletal classes (χ^2 ^3.55, p = 0.17). However, when sex stratification was performed the analysis indicated that females displayed a different distribution among the three facial skeletal classes (χ^2 ^9.21, p = 0.01; Table 2). In fact, the odds ratio were 2.17 (0.69 – 6.61, 95% confidence interval) and 4.61 (1.36 – 15.27, 95% confidence interval) for those female subjects belonging to the second and the third class, respectively, with respect to the subjects belonging to the first class (see Table 2). Moreover, a linear trend was shown going from the first to the third class (χ^2 ^for linear trend 9.011, p < 0.001).

**Table 2:** Distribution of female individuals among the three different facial skeletal classes

	**I Class ***n (%)*	**II Class ***n (%)*	**III Class ***n (%)*	**Total ***n (%)*
**S**	172 (77.48)	34 (15.31)	16 (7.21)	222 (100)
**NS**	14 (53.84)	6 (23.08)	6 (23.08)	26 (100)
**Total**	186	40	22	248

χ^2 ^= 9.21, p = 0.01.

Subjects were sorted either as potentially successful (S) or potentially not-successful (NS) to manage interpersonal relationships, according to the psychiatric evaluation. Percentages refer to the distribution of either S, or NS subjects among the three different facial skeletal classes.

On the other hand, no significant differences were shown in the distribution of NS male subjects among the three skeletal classes (Table 3).

**Table 3:** Distribution of male individuals among the three different facial skeletal classes

	**I Class ***n (%)*	**II Class ***n (%)*	**III Class ***n (%)*	**Total ***n (%)*
**S**	416 (69.10)	80 (13.29)	106 (17.61)	602 (100)
**NS**	110 (67.07)	22 (13.42)	32 (19.51)	164 (100)
**Total**	526	102	138	766

χ^2 ^= 3.55, p = 0.17.

Subjects were sorted either as potentially successful (S) or potentially not-successful (NS) to manage interpersonal relationships, according to the psychiatric evaluation. Percentages refer to the distribution of either S, or NS subjects among the three different facial skeletal classes.

## Discussion

This study investigated the relationship between the facial skeletal class and the capacity of successfully managing interpersonal relationships in a sample of young Italian adults applying to the military academy of Pozzuoli, Italy. This particular population was chosen because it had the advantage of being highly homogeneous for age, and, possibly, more sensitive to those factors determining social and physical appearance. Moreover, the sample of this study had the advantage of being heterogeneous for geographical provenience and, therefore, more representative of the Italian population with respect to regionally conducted surveys.

On the other hand, it is necessary to clarify that the sample study recruited in this study might not accurately represent the situation of an age-matched general population, since a possible selection bias might occur. In fact, those individuals applying to the military academy might differ from the general population both for facial morphology, psychological characteristics, and possibility to access to specific orthodontic treatments. In order to access to the Military Academy, a high school qualification is required, possibly further skewing the population type and rendering generalizations more difficult.

Moreover, the number of enrolled females is about one third with respect to the other sex, presumably further amplifying the possible selection bias for this specific subgroup, considering the small number of subjects that were recruited. Probably due to cultural reasons, it is only recently that in Italy women started to consider the military career as a suitable one and to be considered as a suitable choice with respect to their male counterpart. This phenomenon might have generated another selection bias involving specifically only the group of enrolled female subjects.

Furthermore, it might be theoretically possible that the psychiatric evaluation itself might be biased due to the different attractiveness of the recruited subjects. In fact, the possibility that the psychiatrists judged more frequently as NS those subjects they perceived as being less beautiful can not be ruled out, although the application of the MMPI-2 test (a self-administered test) should have limited this phenomenon. Last but not least, in order to reduce the complexity of the analyzed parameters in a study enrolling more than 1000 subjects, we did not consider in the investigation the vertical dimensions of the face, which have been also shown to be important in determining the perceived beauty (see Michiels and Sather 1994).

Nevertheless, our data clearly indicate that male subjects display an increased risk with respect to females of being NS but not related to the particular facial skeletal class. The reasons for this specific phenomenon are unclear but might be referred to a possible selection bias. In fact, as already reported, our female population might have been skewed in a different way with respect to the male one. Our results show that female subjects display a lower risk with respect to their counterpart but associated to the facial skeletal structure, and in particular to the facial skeletal class III. A possible explanation of this evidence might be indicated in a hypothetical higher susceptibility of female individuals to physical factors determining beauty, such as the facial skeletal class is (Campbell 2004). As a matter of fact, possibly for cultural reasons, social pressure to be physically attractive might be stronger on female individuals with respect to males, hypothetically resulting in higher vulnerability to develop problems in the capability of managing successfully interpersonal relationships, when not fulfilling completely with the society expectancies and demands (Marcus and Miller 2003).

However, alternative explanations might have possibly generated the observed results. For example, although highly improbable, there might be a theoretical genetic predisposition to develop both a specific facial skeletal class, and a particular psychological profile. Therefore, further investigation on this topic might contribute to confirm our findings and clarify these issues. In order to reduce the bias sources, particular attention should be reserved to the recruitment of an appropriate study population and we are now considering the possibility of assessing in future studies the facial characteristics with more complex measures, possibly including vertical features as well.

## Conclusion

Considering the reported findings, we would like to conclude by suggesting that improvements on the facial region by surgical/orthodontic early intervention might theoretically have a significant beneficial impact on the capacity of managing successfully interpersonal relationships in young adults, especially when considering female subjects since, possibly, more sensitive to this issue. Moreover, this study might possibly contribute to understand the structural determinants of the perceived beauty, which might be helpful when planning specific surgical and/or orthodontic intervention.

## Competing interests

The author(s) declare that they have no competing interests.

## Authors' contributions

**AS **contributed substantially to the conception and design of the study and carried out the acquisition of data;

**DA **contributed substantially to the conception and design of the study;

**LT **participated in the design of the study, performed the statistical analysis and was involved in drafting the manuscript;

**GC **participated in the design of the study, performed the statistical analysis and was involved in drafting the manuscript;

**LS **conceived of the study and participated in its design.

All authors read and approved the final manuscript.

## Pre-publication history

The pre-publication history for this paper can be accessed here:


